# Differential Urinary Proteome Analysis for Predicting Prognosis in Type 2 Diabetes Patients with and without Renal Dysfunction

**DOI:** 10.3390/ijms21124236

**Published:** 2020-06-14

**Authors:** Hee-Sung Ahn, Jong Ho Kim, Hwangkyo Jeong, Jiyoung Yu, Jeonghun Yeom, Sang Heon Song, Sang Soo Kim, In Joo Kim, Kyunggon Kim

**Affiliations:** 1 Asan Institute for Life Sciences, Asan Medical Center, Seoul 05505, Korea; zaulim3@gmail.com (H.-S.A.); yujiyoung202@gmail.com (J.Y.); 2 Department of Internal Medicine and Biomedical Research Institute, Pusan National University Hospital, Busan 49241, Korea; bedaya@hanmail.net (J.H.K.); shsong0209@gmail.com (S.H.S.); drsskim7@gmail.com (S.S.K.); 3 Department of Biomedical Sciences, University of Ulsan College of Medicine, Seoul 05505, Korea; hkyo723@naver.com; 4 Convergence Medicine Research Center, Asan Institute for Life Sciences, Seoul 05505, Korea; nature8309@gmail.com; 5 Clinical Proteomics Core Laboratory, Convergence Medicine Research Center, Asan Medical Center, Seoul 05505, Korea; 6 Bio-Medical Institute of Technology, Asan Medical Center, Seoul 05505, Korea

**Keywords:** urine, diabetic kidney disease, kidney function, proteomics, mass spectrometry, statistical clinical model, machine learning

## Abstract

Renal dysfunction, a major complication of type 2 diabetes, can be predicted from estimated glomerular filtration rate (eGFR) and protein markers such as albumin concentration. Urinary protein biomarkers may be used to monitor or predict patient status. Urine samples were selected from patients enrolled in the retrospective diabetic kidney disease (DKD) study, including 35 with good and 19 with poor prognosis. After removal of albumin and immunoglobulin, the remaining proteins were reduced, alkylated, digested, and analyzed qualitatively and quantitatively with a nano LC-MS platform. Each protein was identified, and its concentration normalized to that of creatinine. A prognostic model of DKD was formulated based on the adjusted quantities of each protein in the two groups. Of 1296 proteins identified in the 54 urine samples, 66 were differentially abundant in the two groups (area under the curve (AUC): *p*-value < 0.05), but none showed significantly better performance than albumin. To improve the predictive power by multivariate analysis, five proteins (ACP2, CTSA, GM2A, MUC1, and SPARCL1) were selected as significant by an AUC-based random forest method. The application of two classifiers—support vector machine and random forest—showed that the multivariate model performed better than univariate analysis of mucin-1 (AUC: 0.935 vs. 0.791) and albumin (AUC: 1.0 vs. 0.722). The urinary proteome can reflect kidney function directly and can predict the prognosis of patients with chronic kidney dysfunction. Classification based on five urinary proteins may better predict the prognosis of DKD patients than urinary albumin concentration or eGFR.

## 1. Introduction

About 30% of people with diabetes develop diabetic kidney disease (DKD), and the spread of diabetes is increasing worldwide [1,2]. Complications of type 2 diabetes (T2D) mainly cause end-stage renal disease, which is related to high heart disease incidence and mortality [2,3]. Early detection and screening of patients at risk for DKD is important, which may reduce the global burden of T2D. 

Because the kidneys filter waste from blood and discharge it as urine, urine can directly reflect kidney function. Unlike plasma, urine can be easily collected non-invasively, with proteins in urine being stable and not vulnerable to sudden degradation [4]. Albuminuria and estimated glomerular filtration rate (eGFR), have been generally used to assess kidney function [2,5]. However, albuminuria is only evaluated after glomerular damage has occurred, and sometimes kidney disease develops before the outbreak of albuminuria [6,7]. Better markers are required to help delay progression to DKD. 

Multiple-biomarker approaches based on proteomics, including urinary proteomics, may overcome the limitations of markers diagnostic for DKD [4,8]. This study was designed to identify a urinary multi-protein panel that could predict progression to DKD in patients with T2D.

## 2. Results

### 2.1. Baseline Characteristics of Clinical Samples Used in the Study

Table 1 summarizes the baseline characteristics of patients in the poor and good prognosis groups. All factors did not differ significantly in the two groups, including sex, age, body mass index (BMI), duration of follow-up, systolic blood pressure (SBP), glycated hemoglobin concentration (HbA1c), lipid profile, percent with diabetic retinopathy, and the percentages treated with RAS inhibitors, anti-hypertensive agents, and lipid-lowering agents (Bonferroni corrected *p*-value > 0.05/17 = 0.0029; Table S1).

### 2.2. Urinary Proteome Analysis for Identification and Label-Free Quantitation 

**Figure 1:**
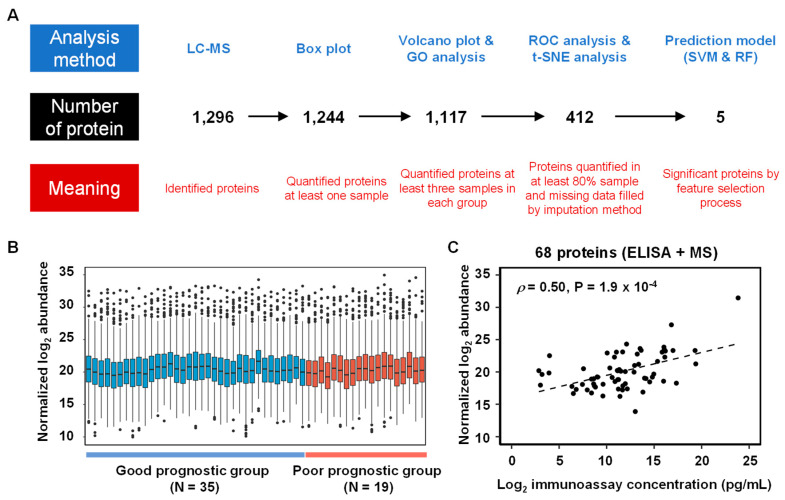
(**A**) Analysis workflow of urinary proteins in the 54 diabetic kidney disease (DKD) patients. The analysis method is written in the upper part, the number of proteins in the middle, and the meaning of the protein in the bottom part. (**B**) Boxplots of normalized urinary protein abundances in the 54 samples (35 patients in good-prognostic group and 19 patients in poor-prognostic group) measured by LC-MS analysis. (**C**) Scatter plot of 68 urine proteins between normalized log2 abundance and log2 immunoassays concentration (Pearson correlation coefficient (*ρ*): 0.5 and *p*-value: 1.9 × 10^−4^).

The workflow of data processes contained identified and quantified urinary proteins indicative of disease status, as well as significant proteins to build the clinical models (Figure 1A). Liquid chromatography–mass spectrometry (LC-MS) analysis of the 54 urine samples identified 1296 proteins (Table S2). Of these proteins, 1244 were quantified, and their quantities were adjusted relative to the concentration of creatinine [9,10]. Sample-to-sample variation was subsequently fixed by the amount of proteins, leading to the selection of six endogenous normalization proteins that showed stable abundance in all LC-MS analyses. A boxplot of protein abundances in the 54 samples, composed of 35 patients in good-prognostic group (GPG) and 19 patients in poor-prognostic group (PPG), is depicted in Figure 1B. The normalized abundance of 68 proteins significantly correlated with their immunoassay [11] determined concentrations in urine, with a Pearson’s coefficient of 0.502 (permutation *p*-value < 0.001; Figure 1C). 

### 2.3. Functional Annotation of Differential Protein Expression in the PPG and GPG Groups 

**Figure 2:**
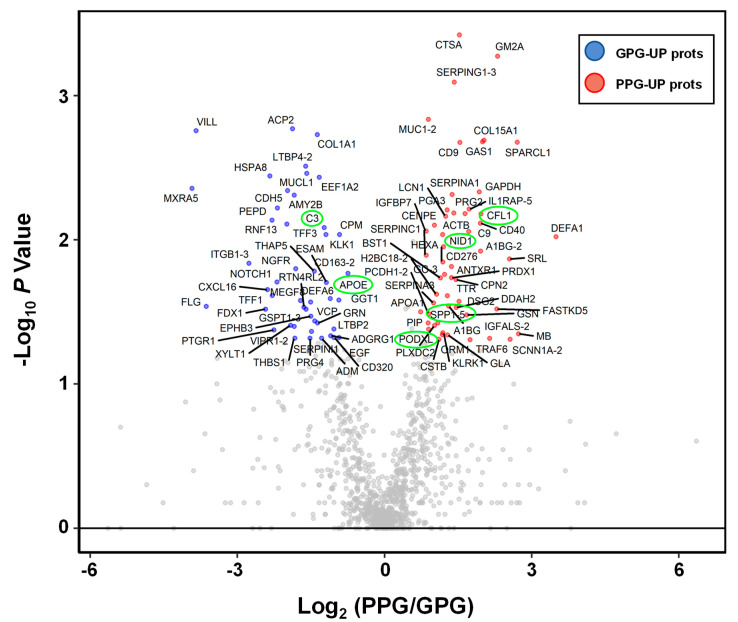
Volcano plot of urinary proteomic data. Volcano plots are depicted with the fold change of each protein abundance and the *p* value was calculated by performing a Mann–Whitney U-test. The averages of the urinary proteomic abundance data of good prognostic group (*N* = 35) were compared with the averages of the data for poor prognostic group (*N* = 19). Red circles show 54 urinary proteins that have significant increases in PPG. Blue circles show 46 urinary proteins which have significant decreases in PPG. Gray circles are urinary proteins without statistical meaning. Green circles are previously released as urinary protein markers for glomerular injury or tubular injury.

To find the differential abundant proteins (DAPs) from among the 1117 proteins, fold-changes and *p*-values were calculated by Mann–Whitney U tests of the two groups. A volcano plot showing log2-fold-changes against minus log_10_ *p*-values identified 46 proteins as being upregulated in the PPG and 54 proteins in the GPG (|log_2_ fold-change| > 0.5; *p*-value < 0.05; Figure 2 and Table S3). These differentially expressed proteins included the six previously described candidate urinary biomarkers (APOE, CO3, COF1, NID1, OSTP-5, and PODXL) of glomerular or tubular injury [11].

To determine whether urinary DAPs were associated with specific biological processes, up- and downregulated proteins in the PPG were subjected to gene ontology (GO) enrichment analysis. To integrate the three domains of GO and easily visualize the relationship between terms, ClueGO tools were applied with default settings (kappa score 0.4 and group merger of 50% of genes) to functionally organize the GO term networks [12].

**Figure 3:**
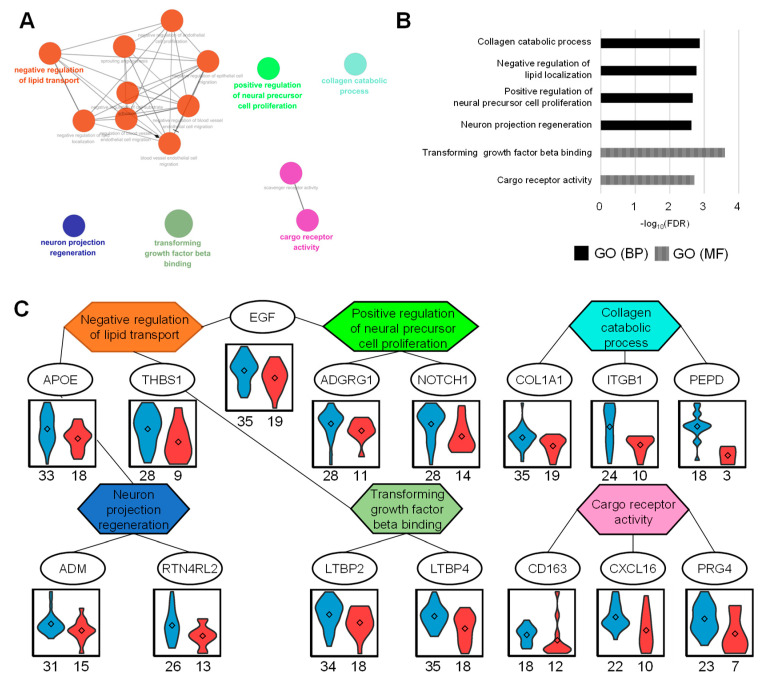
Up-regulated proteome in good prognosis group (GPG) and gene ontology (GO) analysis. (**A**) Functional GO network displaying grouping of GO terms enriched in GPG up-regulated proteins. (**B**) Enriched GO terms in biological process and molecular function. (**C**) The network between GO terms and corresponding proteins represents the relationship between GO terms via the proteins. The abundances of each protein represent the violin plots in two groups. The numbers listed below represent the measured numbers for each group.

Downregulated proteins in PPG were significantly enriched with an FDR < 0.01 (Figure 3A,B). Biological processes associated with these proteins included negative regulation of lipid localization, collagen catabolic process, positive regulation of neural precursor cell proliferation, and neuron projection regeneration. Resulting analysis of molecular function indicated that transforming growth factor beta binding and cargo receptor activity were annotated. The networks between proteins and functional GO terms showed that three proteins were negative regulators of lipid transport, as well as being associated with another GO term (Figure 3C). These included APOE, which is involved in neuron projection regeneration; THBS1, which is involved in transforming growth factor beta binding; and EGF, which is involved in positive regulation of neural precursor cell proliferation. 

**Figure 4:**
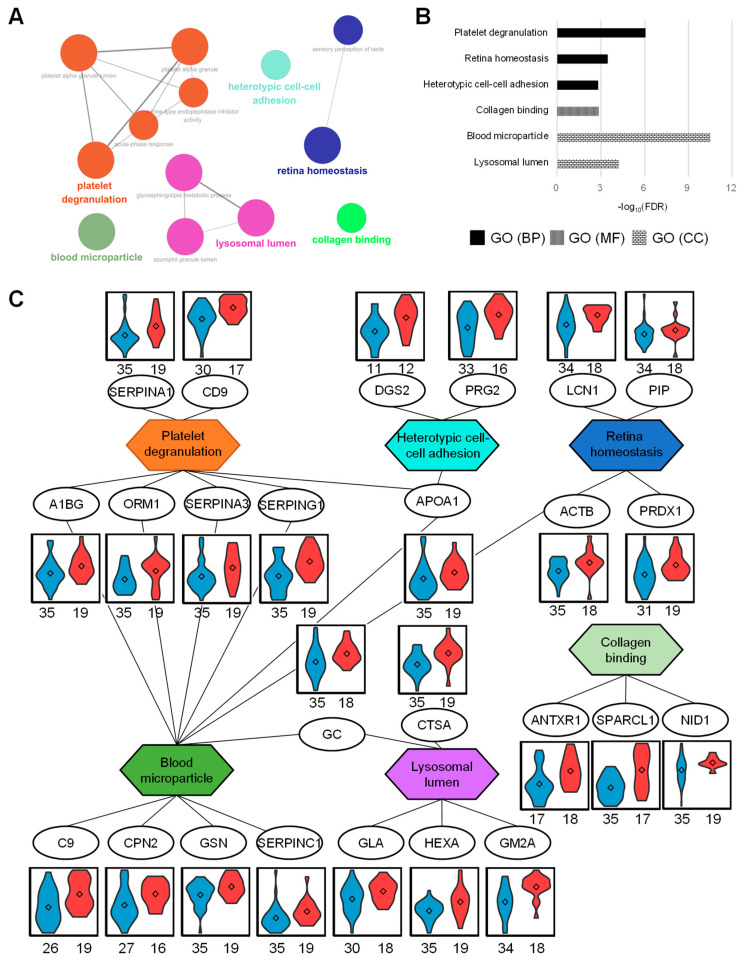
Up-regulated proteome in poor-prognostic group (PPG) and GO analysis. (**A**) Functional GO network displaying grouping of GO terms enriched in PPG up-regulated proteins. (**B**) Enriched GO terms in biological process, molecular function, and cellular component. (**C**) The network between GO terms and their contained proteins represents the relationship between GO terms via the proteins. The abundance of proteins represents the violin plots in two sample groups. The numbers listed below represent the measured numbers for each group.

Upregulated proteins in PPG were identified in enriched functional GO groups with an FDR < 0.01 (Figure 4A,B). Biological processes associated with these proteins included platelet degranulation, retina homeostasis, and heterotypic cell–cell adhesion. The molecular functional processes related with these proteins contained collagen binding. These urinary proteins were located in the lysosomal lumen and blood microparticles. The networks between proteins and functional GO terms indicated that the proteins in blood microparticles were functionally involved in platelet degranulation (Figure 4C). Platelets in patients with CKD are deficient in reactivity [13]. Leukocytes adhere to and destroy damaged kidney cell walls in patients with CKD [14], accompanied by bone marrow-derived kidney fibrosis, which is highly associated with cell–cell adhesion [15]. CKD is also associated with retinal abnormalities [16] and the possible destruction of retinal homeostasis, as confirmed in this study.

### 2.4. Univariate ROC Analysis for Predicting Renal Outcome

**Figure 5:**
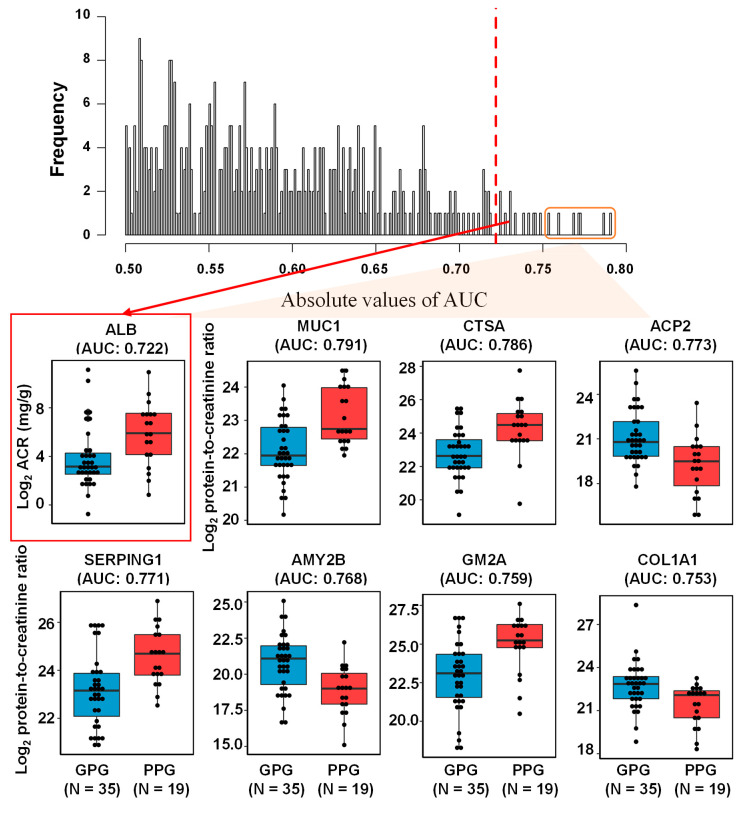
Histogram of area under the ROC curves (AUC) of 412 urinary proteins and ACR. Top seven proteins (MUC1, CTSA, ACP2, SERPING1, AMY2B, GM2A, and COL1A1) and ACR are represented with box plots.

To ensure statistical reliability, this study focused on 412 proteins quantified in more than 80% of urine samples [17], with missing values filled by local least squares imputation [18] (Table S4). To confirm that quantified urinary proteins could act as individual biomarkers, univariate receiver operating characteristic (ROC) analysis was performed in samples from the PPG and GPG, with the resulting histogram of AUC values shown in Figure 5. The AUC values of MUC1, CTSA, ACP2, SERPING1, AMY2B, GM2A, and COL1A1 were 0.791, 0.786, 0.773, 0.771, 0.768, 0.759, and 0.753, respectively. ACP2, AMY2B, and COL1A1 were significantly more abundant, whereas MUC1, CTSA, SERPING1, and GM2A were significantly less abundant, in the GPG than in the PPG (*p*-value < 0.05 each). The 66 urinary proteins showed significance with AUCs of 0.5 (*p*-value < 0.05; Table S5). Clinically, urinary albumin is a common marker of DKD [2,5]. The AUCs of 18 proteins were higher than that of albumin (0.722), but the differences were not statistically significant based on likelihood ratio tests.

### 2.5. Multivariate Analysis for Predicting Renal Outcome

**Figure 6:**
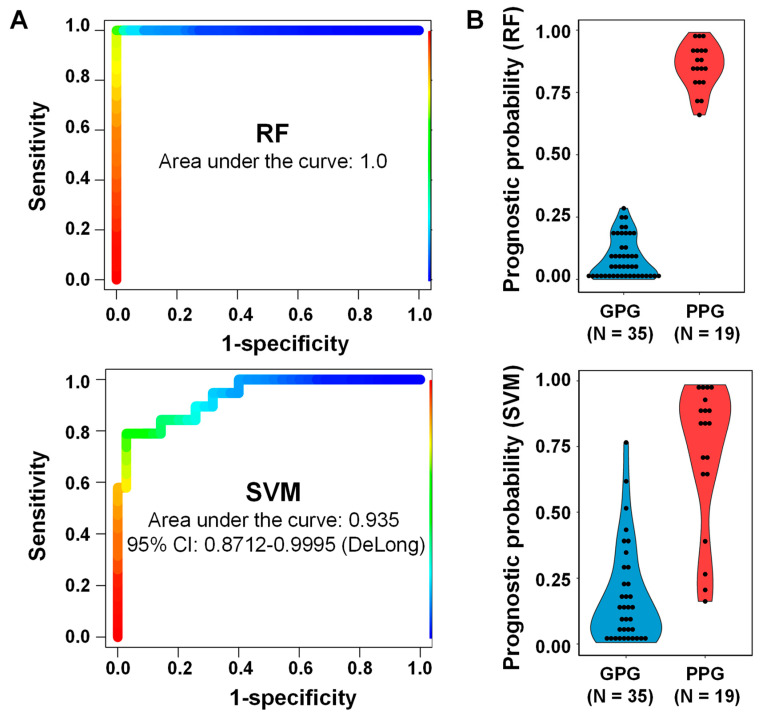
ROC curves of RF and SVM classifiers for five selected proteins (ACP2, CTSA, GM2A, MUC1 and SPARCL1). Performance of the two classifiers in the set of 54 samples, 35 from patients with good prognosis and 19 from patients with poor prognosis. (**A**) Areas under the curve (AUC) for the RF (1.0) and SVM (0.935) classifiers. (**B**) Clinical indices (0–1) of the two classifiers.

To improve predictive performance and find a meaningful combination of proteins that could distinguish patients who were and were not at risk of disease progression, two classifiers of the 412 proteins were generated, one based on random forest (RF) [19] and the other on support vector machine (SVM) [20]. Both the RF and SVM methods selected five proteins (ACP2, CTSA, GM2A, MUC1, and SPARCL1) by an AUC-based RF backward-elimination process [21], according to a >0.3 importance of selection (Table 2). These variables were used to establish a RF model by generating 20,000 decision trees, and a linear SVM model by three repeated iterations of 10-fold cross-validation. Evaluation of the performance of these classifiers showed that the AUC values for RF and SVM were 1.000 and 0.935, respectively (Figure 6A). The nominal binary results of RF and SVM models were transformed in disease prediction scores, which ranged from 0 to 1 (Figure 6B and Table S6). The two classifiers differed significantly from albumin-to-creatinine ratio (likelihood ratio test: *p*-value < 0.05). These five proteins were located in extracellular exosomes, vesicles, or organelles, with three (ACP2, CTSA, and GM2A) located in the lysosomal lumen, MUC1 placed in plasma membrane, and SPARCL1 interacted with collagen in extracellular matrix. 

### 2.6. External Validation of Clinical Models in Public Studies

**Figure 7:**
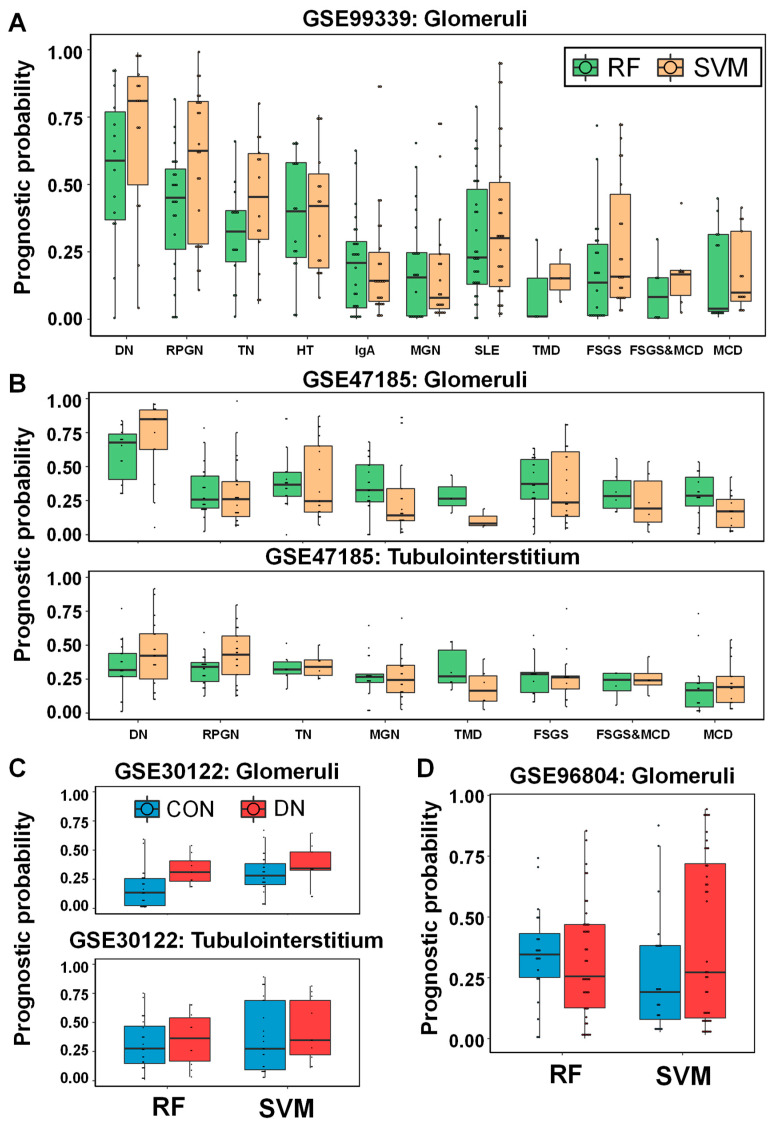
External validation of RF and SVM clinical models in public four GEO datasets (GSE99339, GSE47185, GSE30122 and GSE96804). (**A**) In the GSE99339 dataset, boxplot of the prognostic probabilities of the two classifiers in 11 disease groups including DN (*N* = 14), RPGN (*N* = 23), TN (*N* = 14), HT (*N* = 15), IgA nephropathy (*N* = 26), MGN (*N* = 21), SLE (*N* = 30), TMD (*N* = 3), FSGS (*N* = 22), FSGS&MCD (*N* = 6), and MCD (*N* = 13). (**B**) In the GSE30122 data set, the prognostic indexes of the two classifiers in the eight disease groups in the renal glomeruli with DN (*N* = 14), RPGN, (*N* = 23), TN (*N* = 17), MGN (*N* = 21), TMD (*N* = 3), FSGS (*N* = 23), FSGS&MCD (*N* = 6), and MCD (*N* = 15) and in the renal tubulointerstitia with DN (*N* = 18), RPGN (*N* = 21), TN (*N* = 6), MGN (*N* = 18), TMD (*N* = 6), FSGS (*N* = 13), FSGS&MCD (*N* = 4), and MCD (*N* = 15). (**C**) In the GSE30122 data set, the prediction values of the two classifiers in the control and disease groups in renal glomerulus (*N* = 26; control and *N* = 9; disease) and in renal tubulus (*N* = 24; control and *N* = 10; disease). (**D**) In the GSE30122 data set, the prediction probabilities of the two classifiers in the control (*N* = 20) and disease (*N* = 41) groups in renal glomeruli.

Since we were unable to find a benchmarking study in the discovery of urine protein biomarkers that could validate our statistical model, we validated the models with mRNA expression in the kidney, an organ that undoubtedly affects urine samples. The SVM and RF models consisting of five urine proteins were applied to four publicly available GEO datasets (GSE99339 [22], GSE47185 [23], GSE30122 [24], and GSE96804 [25,26]) without model adjustment. In the first GSE99339 dataset, mRNA expression in the renal glomerulus of 187 patients was studied, and the 11 disease groups are diabetic nephropathy (DN), rapidly progressive glomerulonephritis (RPGN), tumor nephrectomies (TN), hypertensive nephropathy (HT), IgA nephropathy, membranous glomerulonephritis (MGN), systemic lupus erythematosus (SLE), thin membrane disease (TMD), focal and segmental glomerulosclerosis (FSGS), focal and segmental glomerulosclerosis and minimal change disease (FSGS&MCD), and minimal change disease (MCD). The two classifiers’ prognostic probabilities were highly correlated with each other in 187 samples (*ρ* = 0.817, Pearson correlation coefficient). In both models, the highest value in the DN group was higher than the other ten disease groups (Figure 7A). RF prediction values in the DN group were significantly higher than other eight groups except for RPGN and HT group (Mann-Whitney U Test: *p*-value < 0.05). SVM prediction values in the DN group were significantly higher than the other nine groups excluding the RPGN group (*p*-value < 0.05). In the second GSE99339 data set, there are a total of 223 kidney glomerulus (*N* = 122) and tubulointerstitia (*N* = 101) mRNA expression levels. The eight disease groups include DN, RPGN, TN, MGN, TMD, FSGS, FSGS&MCD, and MCD. The two classifiers’ prognostic probabilities were also highly correlated in 223 samples (*r* = 0.637). The tendency of the predicted values was different depending on the cell type of the kidney (Figure 7B). In the glomeruli, the two model predictions are the highest in the DN group and are statistically significant with other seven groups. However, in the tubulointerstitium, the SVM model prediction values in DN were significant with four other groups except RPGN, TN, FSGS&MCD, and RF model prediction values in DN is only significant with MCD. It indicated that the five urine proteins are more closely related to the glomeruli than the kidney tubulointerstitium. 

Meanwhile, we tried to verify whether the prognostic models could predict DKD. In the third GSE30122 data set, of the total of 69 samples, 26 of the 35 kidney glomerulus were normal obtained from living allograft donors, 9 of which were DKD, 34 of which were renal tubulus, of which 24 were normal and 10 were DKD. The results of the RF model in the glomeruli statistically were divided the normal and disease groups (*p*-value < 0.05), but the SVM model were not (*p*-value > 0.05; Figure 7C). The results of the both models in the tubulus statistically were not divided the normal and disease groups (*p*-value > 0.05). In the fourth GSE30122 dataset, 20 kidney glomerulus out of a total of 62 samples were glomerulus from the non-neoplastic part of tumor nephrectomies and 41 of them were from DN. The results of the both models statistically were not divided the normal and disease groups (*p*-value > 0.05; Figure 7D). It indicated that models for predicting kidney prognosis with urine protein markers in diabetics are difficult to distinguish DKD from normal groups by mRNA expression level in kidney.

## 3. Discussion

Urine-based approaches for measuring internal biomolecules can be normalized. Ideally, urine should be collected for 24 h and urinary biomolecules measured. Because this method is practically difficult, urinary proteins in random spot samples were calibrated relative to creatinine concentrations [9]. Prolonged storage of urine samples for studying proteins is important because of the activity of urinary proteases depending on the temperature and pH [27]. In this retrospective study, urine samples were stored at −80 °C for 7–8 years before LC-MS/MS measurements. In general, it is known that it is stable without urine preservatives stored at −70 or −80 °C, and urine samples stored for more than 2.3 years have no significant change in not only most proteins including albumin but also metabolites including creatinine [28,29,30,31].

Proteins were extracted from urine samples using an equal volume-based approach similar to ELISA [32]. This procedure for protein standardization was suitable for downstream analysis. Urinary proteins normalized by this method showed lower sample-to-sample variation and higher correlation with immunoassay results.

Albuminuria is primarily used to detect DKD in clinical practice [2,5]. Because glomeruli filter blood, albumin is a good biomarker of chronic kidney disease (CKD) caused by glomerular abnormalities but is insufficient to determine subsequent prognosis [5]. Rather than this, it was determined that finding and measuring specific protein markers that affect pathological function is more clinically meaningful [33,34]. Although causality between albuminuria and prognostic values from the five-protein panel-based clinical models (RF and SVM) cannot be clarified in this retrospective study, it can be inferred by correlation analysis. Correlation analysis between two classifiers and ACR in the 54 enrolled patients reveals a little of bit correlation but no significance (*r* = 0.086; *p*-value > 0.05; SVM and *r* = 0.094; *p*-value > 0.05; RF). Therefore, it was confirmed that there was no causal relationship as well as a correlation. To consider closely at the relationship between them, we divided the three classes based on the ACR value (normal; <30 mg/g, microalbuminuria; 30–300 mg/g and macroalbuminuria; >300 mg/g) and plotted the predicted values of the SVM model according to the two prognostic groups (Figure S1). In T2D patients with normal range and microalbuminuria, SVM results were almost separated between two groups. Rather, it seems to have problems with predictive power in patients with macroalbuminuria. It means that SVM results did not depend on the development of albuminuria in T2D patients and showed the possibility to predict the earlier disease stage before the development of albuminuria. Moreover, the predicted value of RF results accurately separates two prognostic groups regardless of the ACR value.

As a rule, diabetics are persistently exposed to miscellaneous metabolic and hemodynamic risks [35], with DKD resulting from multiple pathophysiological processes. Multiple-biomarker approaches using proteomics and metabolomics may better reveal the complicated disease status thought to be associated with the onset of DKD [4,8]. CKD273, a panel consisting of 273 urinary peptides currently undergoing Phase 3 testing, was a high performance urine peptidomic classifier for CKD diagnosis [36]. Moreover, this classifier was recently validated as a predictor of the development of microalbuminuria in normoalbuminuric with diabetic patients [37]. These 273 intact peptides were derived from 30 independent proteins, 24 of which were quantified in this study. CKD273, which includes cleaved collagenase peptides and SERPINA1 peptides, is a good prognostic marker, showing that the concentrations of cleaved collagenase peptides decrease and those of SERPINA1 peptides increase in the urine of patients with CKD [38,39]. The present study showed a similar pattern of abundance in the urine of PPG patients despite artificial digestion. Our approach, based on protein concentrations in urine samples, could better explain the pathological pathway associated with DKD than the peptidome approach. Indicators of kidney dysfunction include increased blood particles in urine; lysosomal dysfunction in glomerular cells [40], which is related to the autophagy-lysosome pathway [41] abnormal heterotypic cell–cell adhesion among glomerular, tubular, and immune cell compartments, collagenase, and binding proteins (driven by rapid changes in glycolipids) [42] and platelet activation [43]. 

Our clinical models consist of five selected proteins, four proteins (CTSA, MUC1, GM2A, and SPARCL1) are high in PPG and other one protein (ACP2) is low in PPG. Cystatin A (CTSA), in the same protein family as cystatin C that can measure kidney function [44,45,46], has been found in our urinary biomarker discovery study. Muc1 is a multifaceted tumor protein, and its relationship with the kidney has recently been highlighted [47] and has been identified as a mutant that causes mendelian disorder medullary cystic kidney disease type 1 [48]. In a meta-analysis study of rat glomerular transcriptome profiling, it was confirmed that GM2A was highly expressed in various diabetic kidney disease rat [49]. Through the mouse kidney injury model experiment, SPARCL1 showed that mRNA expression was not changed in the acute phase, but the expression level was high in the fibrosis of the kidney [50], and it inhibited the movement and invasion of renal cell carcinoma [51]. Lastly, ACP 2, one of the lysosomal enzymes, is a protein used in peptiduria [52] or lysosomal enzymuria [53] that measures kidney disease in diabetic patients. Through the external kidney mRNA published studies, these urine biomarkers we found confirmed differential expression in kidney tissue with DKD.

This study had several limitations. First, the patient population in this study was homogeneous and of small sample size. These results require further validation in a multiethnic cohort including larger numbers of patients to assess applicability to a wider population with T2D, a study currently in progress. Second, DKD was clinically diagnosed in the absence of renal biopsies. Third, it is unclear which organ is derived from the urinary protein signatures. More research is needed to determine whether urinary protein signatures are biomarkers of tubular damage in pathological conditions with a glomerular protein load.

## 4. Materials and Methods 

### 4.1. Patients and Urine Samples 

Urine samples were collected from 54 outpatients with T2D and eGFR ≥ 60 mL/min/1.72 m^2^ who were enrolled in the DKD study at Pusan National University Hospital, South Korea, from February 2010 to January 2011 and who met previously described inclusion and exclusion criteria [54]. After one year, patients were followed-up with until September 2017. Patients were managed according to standard guidelines, including treatment with RAS inhibitors, and eGFR was measured at least twice during a follow-up period ≥12 months. Renal function decline was defined as an eGFR < 60 mL/min/1.72 m^2^, annual eGFR reduction > 3 mL/min/1.72 m^2^, or CKD progression, defined as a reduction in GFR category, accompanied by a ≥ 25% deterioration in eGFR from baseline. The patients were divided into two groups—19 with renal outcomes (poor prognosis group (PPG)) and 35 without renal outcomes (good prognosis group (GPG)). The protocols and consent procedures were approved by the Institutional Review Board of Pusan National University Hospital (approval No. 2013033). Total proteinuria and albuminuria, as well as creatinine concentrations, were measured in random spot urine samples [55].

### 4.2. Measurements of Nephrology Parameters

eGFR was calculated using the equation eGFR = 141 × min (serum creatinine/kappa, 1) alpha × max (serum creatinine/kappa, 1) − 1.209 × 0.993 × age × sex × race. For females, sex = 1.018; alpha = −0.329; and kappa = 0.7; for males, sex = 1; alpha = −0.411; and kappa = 0.9. Renal outcomes were chronic kidney disease (CKD) progression based on guidelines of the International Society of Nephrology; accelerated eGFR decline, defined as an annual eGFR reduction > 3 mL/min/1.72 m^2^; or the development of CKD stage ≥ 3. CKD stages 1, 2, 3a, 3b, 4, and 5 were defined as eGFRs of ≥ 90, 60–89, 45–59, 30–44, 15–29, and < 15 mL/min/1.73 m^2^, respectively, and CKD progression was defined as a decline in eGFR category accompanied by a ≥ 25% deterioration in eGFR from baseline [56]. 

### 4.3. Urinary Protein Sample Preparation

Urine samples were centrifuged at 13,000 rpm for 30 min to remove debris, and 300 µL of each supernatant was mixed with 100 µL High Select™ HSA/Immunoglobulin Depletion Resin (Cat. No: A36368, Thermo Fisher Scientific, Waltham, MA, USA) and incubated for 1 h at 4 °C to remove albumin and immunoglobulin. Following centrifugation at 13,000 rpm for 10 min, the supernatant was dried using a speed vac with a cold trap (CentriVap Cold Traps, Labconco, Kansas City, MO, USA). 

### 4.4. Enzymatic Digestion in-Solution

Each dried urine sample was dissolved in 100 µL of 8 M urea, reduced with 20 mM dithiothreitol in 50 mM NH_4_HCO_3_ for 60 min at 25 °C, and alkylated with 40 mM iodoacetamide in 50 mM NH4HCO3 for 60 min in the dark. Urea concentration was diluted to less than 1.0 M. Each urine sample was incubated overnight at 37 °C with 12.5 µg sequencing grade modified trypsin/LysC (Promega, Madison, WI, USA) in 50 mM NH4HCO3 buffer (pH 7.8), followed by quenching with 10uL of 5% formic acid and lyophilization with a cold trap. The samples were re-suspended in 0.1% formic acid, desalted using C18 ZipTips (Millipore, Burlington, MA, USA), and dried for LC-MS analysis.

### 4.5. Nano-LC-ESI-MS/MS Analysis

Digested peptides were separated using a Dionex UltiMate 3000 RSLCnano system (Thermo Fisher Scientific, Waltham, MA, USA). Tryptic peptides from the bead column were reconstituted in 100 μL of 0.1% formic acid and separated on an Acclaim™ Pepmap 100 C18 column (500 mm × 75 μm i.d., 3 μm, 100 Å) equipped with a C18 Pepmap trap column (20 mm × 100 μm i.d., 5 μm, 100 Å; Thermo Fisher Scientific, Waltham, MA, USA) over 200 min (250 nL/min) using a 0–48% acetonitrile gradient in 0.1% formic acid and 5% DMSO for 150 min at 50 °C. The LC was coupled to a Q Exactive™ Plus Hybrid Quadrupole-Orbitrap™ mass spectrometer with a nano-ESI source. Mass spectra were acquired in a data-dependent mode with an automatic switch between a full scan and 10 data-dependent MS/MS scans. The target value for the full scan MS spectra, selected from a 350 to 1800 *m*/*z*, was 3,000,000 with a maximum injection time of 50 ms and a resolution of 70,000 at *m*/*z* 400. The selected ions were fragmented by higher-energy collisional dissociation in the following parameters: 2 Da precursor ion isolation window and 27% normalized collision energy. The ion target value for MS/MS was set to 1,000,000 with a maximum injection time of 100 ms and a resolution of 17,500 at *m*/*z* 400. Repeated peptides were dynamically excluded for 20 s. All MS data were measured once per sample and have been deposited in the PRIDE archive (www.ebi.ac.uk/pride/archive/projects/PXD016571) [57] under Project 1-20191129-77373 12.

### 4.6. Database Searching and Label-Free Quantitation

The SwissProt human database (May 2017) was searched for acquired MS/MS spectra using SequestHT on Proteome discoverer (version 2.2, Thermo Fisher Scientific, USA) [58]. The search parameters were set as default including cysteine carbamidomethylation as a fixed modification, and N-terminal acetylation and methionine oxidation as variable modifications with two miscleavages. Peptides were identified based on a search with an initial mass deviation of the precursor ion of up to 10 ppm, with the allowed fragment mass deviation set to 20 ppm. When assigning proteins to peptides, both unique and razor peptides were used. Label-free quantitation (LFQ) was performed using peak intensity for unique peptides of each protein. 

### 4.7. Normalization of Protein Abundance

To correct for sampling variations resulting from random spot urine collection, the raw LFQ values for each protein were divided by the amounts of total protein and creatinine in each sample, followed by normalization of the corrected LFQ values by endogenous proteins without spike-in standards [59]. To identify endogenous urinary proteins for normalization, the 112 initial completely quantified proteins were considered, with six selected based on the following criteria: (1) quantified in all 54 samples; (2) corrected LFQ values did not differ significantly in the poor and good prognosis groups by the Mann–Whitney U Test (*p*-value > 0.05); and (3) had nearly persistent urine concentrations throughout the sample as top-ranked by NormFinder stability value [60]. 

The corrected LFQ values of the six selected normalization proteins in each sample were divided by their median value in all samples. The median of these six ratios was defined as the normalization scaling factor (NSF) for that sample. For example, NSF for sample s can be determined using the equation:

(1) $$NSF_{s}=median\left(\frac{N_{1,s}}{{\hat{N}}_{1}},\;\frac{N_{2,s}}{{\hat{N}}_{2}},\;\cdots ,\;\frac{N_{6,s}}{{\hat{N}}_{6}}\right)$$

where $N_{i,\;s}$ is the corrected LFQ value of normalization protein i in sample s and $\hat{N_{i}}$ is the median corrected LFQ value of normalization protein i in all samples. Except for the six normalization proteins in a sample, the normalized LFQ value of each protein was calculated by dividing its corrected LFQ value by NSF.

(2) $$\overset{˘}{LFQ_{j,s}}=\frac{LFQ_{j,\;s}}{NSF_{s}}$$

where $\overset{˘}{LFQ_{j,s}}$ is the normalized LFQ of urinary protein j in sample s and $LFQ_{j,s}$ is the corrected LFQ of the corresponding protein [61]. 

### 4.8. Differential Data Analysis by Filling Missing Data

For clinical utility, the LFQ data were filtered to <20% of quantified proteins in each sample group to analyze the differential urinary proteins in these groups, with the missing data filled by the local least squared imputation method at the normalized abundance [18]. 

### 4.9. GO Analysis

Differential abundant proteins (DAPs) in the poor and good prognosis groups were analyzed using the ClueGO (version 2.5.1) [12] plugin for Cytoscape (version 3.6.1) [62]. To group GO terms, the kappa score was set at 0.4 and the number of overlapping genes to combine groups was set at 50%.

### 4.10. Statistical Clinical Model Generation Based on Feature Selection

The process of feature selection was to find the best subset for classifying two disease progression groups out of 412 proteins. There are two steps. In the first step, 50,000 decision trees containing eight variables were randomly generated 50,000 trees and had AUC values. Based on the AUCs values, the optimal number of proteins were determined by out-of-bag error estimation and the value is 11. Second, through the 100 iterations with three-fold cross-validation for from the selected 11 optimal variables, the probability and importance that each variable was included in the model was calculated. We selected five proteins (>0.3 importance). Prior to model building, centering and scaling were performed as preprocessing on the data. In the clinical models, SVM model with linear kernel was generated by a 10 repeated three-fold cross validation method (parameter C = 0.1052) and The RF model was made by a three-fold cross validation method repeated 100 times with 1000 trees, mtry = 5 and nodesize = 5.

### 4.11. Mining Public Microarray Data

We downloaded the mRNA expression data (series accession number: GSE99339, GSE47185, GSE30122, and GSE96804) in the Gene Expression Omnibus database [63]. Then, using GEO2R interactive web tool, five identifiers matching the five selected genes according to the platform record and their expression values were extracted.

### 4.12. Statistical Analysis

Data were analyzed using RStudio (version 1.1.456) including R (version 3.6.0). Statistical R software packages included ggplot2 for drawing box, scattering, volcano and violin plots, permcor for calculating permutation-based *p*-values for Pearson correlation [64], pcaMethods for missing value estimation [65], pROC for univariate ROC analysis [66], ROCR for multivariate ROC analysis, AUCRF for feature selection [21], caret for building statistical classifiers [67], randomForest for building a RF classifier, and e1071 for building a SVM classifier. 

## 5. Conclusions

These results suggest that measurement of urinary proteome was more promising than albuminuria alone for predicting renal outcomes in patients with type 2 diabetes. A panel of five proteins had the potential for use as a biomarker in clinical practice.

## Figures and Tables

**Table 1:** Baseline characteristics of the patients with type 2 diabetes (T2D) with and without renal outcomes.

Variable	With Renal Outcome	Without Renal Outcome
Sex, *n* (%)		
Male	8 (42.1)	11 (31.4)
Female	11 (57.9)	24 (68.6)
Age at diagnosis of diabetic kidney disease, mean ± SD (years)	54.58 ± 11.66	58.66 ± 9.19
BMI, mean ± SD (kg/m^2^)	22.64 ± 3.46	23.81 ± 3.00
Duration of follow-up, mean ± SD (years)	4.80 ± 1.96	4.73 ± 1.94
SBP, mean ± SD (mmHg)	126.58 ± 15.70	125.97 ± 12.07
LDL cholesterol, mean ± SD (mg/dL)	104.89 ± 41.00	99.83 ± 32.32
HDL cholesterol, mean ± SD (mg/dL)	48.42 ± 7.50	52.51 ± 11.51
Triglycerides, mean ± SD (mg/dL)	145.74 ± 99.80	154.57 ± 128.88
eGFR after 1 years, mean ± SD (mL/min/1.73 m^2^)	91.52 ± 17.57	88.33 ± 15.46
HbA1c, mean ± SD (%)	8.34 ± 2.09	7.16 ± 1.36
ACR, mean ± SD (mg/g)	213.66 ± 446.75	126.11 ± 419.70
NAPCR, mean ± SD (mg/g)	178.18 ± 209.30	154.76 ± 299.68
PCR, mean ± SD (mg/g)	391.84 ± 652.79	280.87 ± 711.04
Diabetic retinopathy, *n* (%)	9 (47.37)	11 (31.43)
RAS inhibitor, *n* (%)	6 (31.58)	15 (42.86)
Anti-hypertensive agent, *n* (%)	5 (26.32)	12 (34.29)
Lipid-lowering agent, *n* (%)	10 (52.63)	20 (57.14)

Abbreviations: BMI, body mass index; SBP, systolic blood pressure; LDL, low-density lipoprotein; HDL, high-density lipoprotein; eGFR, estimated glomerular filtration rate; HbA1c, glycated hemoglobin; ACR, urine albumin-to-creatinine ratio; NAPCR, urine nonalbumin protein-to-creatinine ratio; PCR, urine protein-to-creatinine ratio.

**Table 2:** AUC-based RF backward-elimination process-based selected feature proteins.

Uniprot Accession No.	Gene Name	Importance	Prob. Select	Selection	Univariate AUC
P10619	*CTSA*	0.422	0.700	Y	0.737
Q14515	*SPARCL1*	0.378	0.583	Y	0.659
P17900	*GM2A*	0.373	0.613	Y	0.726
P15941-2	*MUC1*	0.332	0.543	Y	0.791
P11117	*ACP2*	0.312	0.563	Y	0.718
P19961	*AMY2B*	0.299	0.510	N	0.779
P00734	*F2*	0.296	0.483	N	0.694
P06865	*HEXA*	0.274	0.466	N	0.651
P05155-3	*SERPING1*	0.275	0.377	N	0.771
P11142	*HSPA8*	0.238	0.330	N	0.734
P10451	*SPP1*	0.228	0.323	N	0.680

Probability of selection for each variable.

## Supplementary Materials

Supplementary materials can be found at https://www.mdpi.com/1422-0067/21/12/4236/s1. Figure S1. Prognostic probability of 54 patients by RF and SVM classifier divided into three groups by albumin-to-creatinine ratio.; Table S1. Demographic and clinical characteristics of the 54 patients with type 2 diabetes.; Table S2. Search result of MS/MS spectra of protein sequences obtained from the 54 urine samples in the Human Swissprot proteome database using the SequestHT search engine.; Table S3. Results of volcano plot analysis.; Table S4. Normalized abundance of 412 urinary proteins in the 54 patients with type 2 diabetes.; Table S5. Univariate receiver operating curve analysis of the areas under the curves of 412 urinary proteins.; Table S6. Prognostic probability by RF and SVM classifier of 54 patients.

## Abbreviations

T2D	Type 2 diabetes
eGFR	Estimated glomerular filtration rate
DKD	Diabetic kidney disease
BMI	Body mass index
SBP	Systolic blood pressure
HbA1c	Glycated hemoglobin concentration
LDL	Low-density lipoprotein
HDL	High-density lipoprotein
ACR	Albumin-to-creatinine ratio
NAPCR	Nonalbumin protein-to-creatinine ratio
PCR	Urine protein-to-creatinine ratio
GPG	Good-prognostic group
PPG	Poor-prognostic group
LC-MS	Liquid chromatography–mass spectrometry
DAP	Differential abundant protein
GO	Gene ontology
FDR	False discovery rate
ROC	Receiver operating characteristic
AUC	Area under the receiver operating characteristic curve
RF	Random forest
SVM	Support vector machine
ELISA	Enzyme-linked immunosorbent assay
CKD	Chronic kidney disease
MS/MS	Tandem mass spectrometry
LFQ	Label-free quantitation
NSF	Normalization scaling factor
DN	Diabetic nephropathy
FSGS	Focal and segmental glomerulosclerosis
FSGS&MCD	Focal and segmental glomerulosclerosis and minimal change disease
HT	Hypertensive nephropathy
MCD	Minimal change disease
MGN	Membranous glomerulonephritis
RPGN	Rapidly progressive glomerulonephritis
SLE	Systemic lupus erythematosus
TMD	Thin membrane disease
TN	Tumor nephrectomies
