# Supplementary material for: Differential Urinary Proteome Analysis for Predicting Prognosis in Type 2 Diabetes Patients with and without Renal Dysfunction
Source: Int J Mol Sci. 2020 Jun 14;21(12):4236. doi: 10.3390/ijms21124236 (PMC7352871; doi:10.3390/ijms21124236)
Supplement: Supplementary file 1 [file ijms-21-04236-s001.zip › 2nd_revised_supplementary_materials/Supplementary Figure.pptx]

## Slide 1
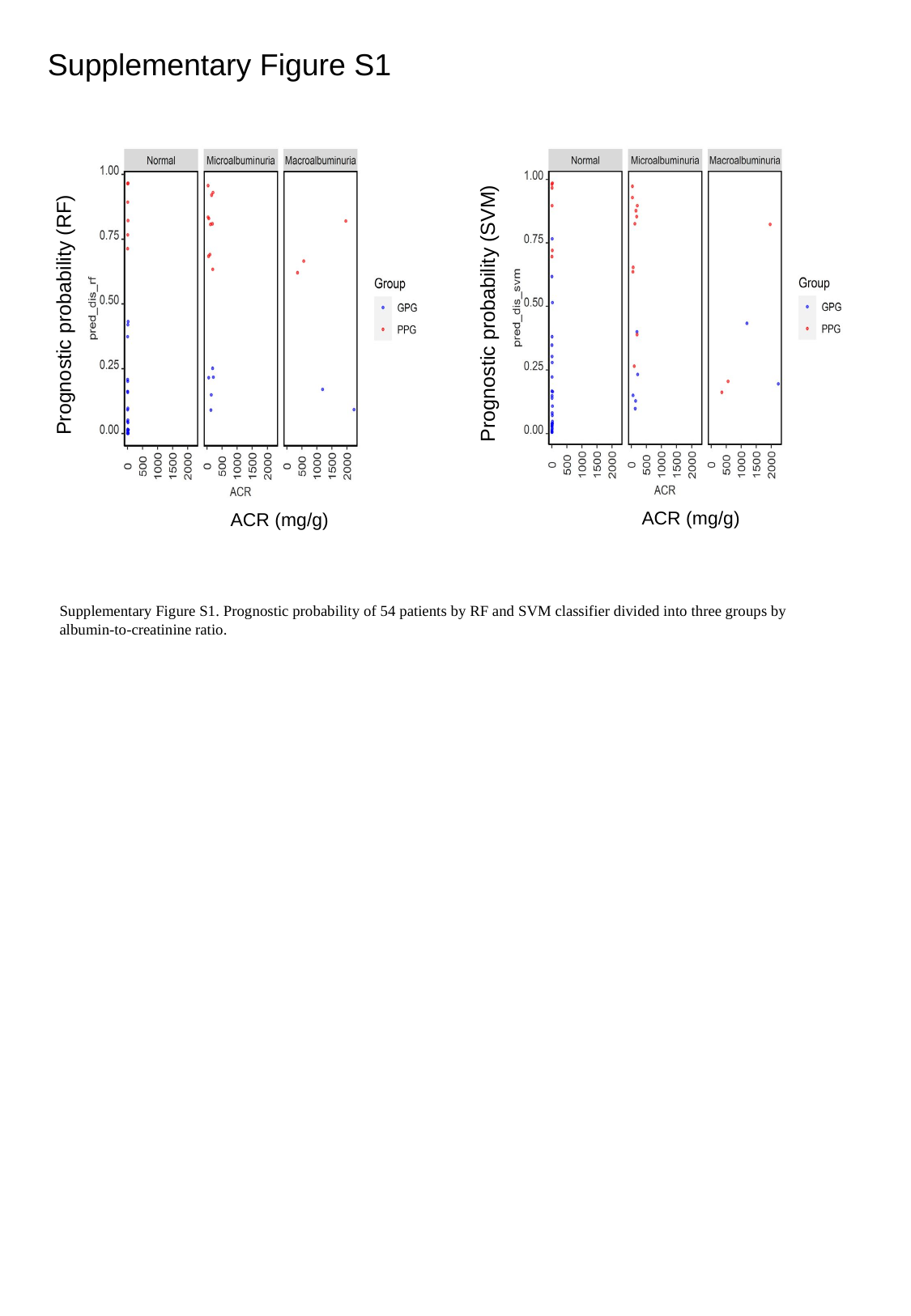

Supplementary Figure S1
Prognostic probability (RF)
ACR (mg/g)
Prognostic probability (SVM)
ACR (mg/g)
Supplementary Figure S1. Prognostic probability of 54 patients by RF and SVM classifier divided into three groups by albumin-to-creatinine ratio.
